# Locomotion Score and Postpartum Conception in Jersey Cows Raised under Hot–Humid Tropical Conditions: A Prospective Study

**DOI:** 10.3390/vetsci11030102

**Published:** 2024-02-27

**Authors:** Karina Vilés, Andrés García, Octavio Rugel, Nahim Jorgge

**Affiliations:** 1 Department of Animal Production, Faculty of Veterinary Medicine and Zootechnics, Agrarian University of Ecuador, Guayaquil 090104, Ecuador; andresgarciaq21@outlook.com (A.G.); drugel@uagraria.edu.ec (O.R.); njorgge@uagraria.edu.ec (N.J.); 2 National Network of Natural Protected Areas of the Civil Society (RESNATUR), Bogota 111311, Colombia; 3 Faculty of Economic Sciences and Business, ECOTEC University, Guayaquil 092302, Ecuador

**Keywords:** dairy cattle, tropics, lameness, reproductive performance

## Abstract

**Simple Summary:**

Rearing livestock in tropical conditions is a crucial challenge for sustainable production in equatorial countries, where cattle are more exposed to more extreme environmental conditions than those of their origin. Jersey milking cows raised under hot–humid tropical conditions were scored for lameness, and the results were related to postpartum conception. Lameness affected reproductive performance, since more than double the number of inseminations was required to conceive, increasing the days open and decreasing the herd’s conception rate. Systematic locomotion scoring represents a fundamental routine procedure to maintain a healthy herd in terms of both foot health and reproductive performance in tropical dairy herds, where environmental conditions greatly worsen common production diseases such as lameness.

**Abstract:**

Reproductive physiology is one of the first systems which is altered when an animal suffers from an imbalance. This is crucial in tropical dairy farming, where maintaining homeostasis and production is particularly demanding. Lameness is a disorder commonly identified by impaired walking, but its early diagnosis could reduce the negative repercussions on production, welfare, and postpartum conception. To evaluate the effect of lameness on postpartum conception, a prospective observational cohort study with a cross-sectional design was developed. Fifty-two Jersey milking cows raised under hot–humid tropical conditions were scored using a five-point locomotion scoring (LS) system (1—non-lame, 2—slightly lame, 3—moderately lame, 4—lame, and 5—severely lame), considering scores ≥ 3 to indicate clinical lameness. Inseminations per conception and days open (CCI) were registered. Inseminations were similar in animals scoring 1, 2, 3, and 5, while they increased in cows with a score of 4, which also increased their CCI along with animals that scored 5. Positive correlations were observed between LS and reproductive variables. The herd’s conception rate was reduced from 45% to 21.8% in the presence of clinical lameness (score ≥ 3). Applying the LS system should be essential as part of routine medical examinations used to monitor dairy herds, and it becomes even more crucial under hot–humid tropical environments, where adverse conditions could rapidly aggravate the early stages of lameness and not only increase the costs of hoof care, but also delay fertility in cattle.

## 1. Introduction

Lameness is usually defined as the manifestation of painful disorders affecting the locomotor system, resulting in changes in movement or deviation from normal posture and/or gait. Its clinical manifestation depends on severity, which ranges from stiffness or decreased symmetry of limb movement to the inability to bear weight on one limb, or, in extreme cases, it can even result in full recline [1,2,3]. However, subclinical disorders do not necessarily cause such changes [4]. It is considered the third-costliest health problem after reduced fertility and mastitis, which result in the culling of dairy cattle [5,6,7]. Due to this, the importance of lameness regarding health and profitability has been considered a main concern for years, since early diagnosis, prevention, and treatment decrease the related direct and indirect costs [6,7,8]. Nonetheless, lameness has recently received considerable attention in terms of welfare because it represents one of the most pressing issues related to welfare due to the associated discomfort, pain, and reduced ability to perform essential natural behaviours for life, such as feeding, rumination, and resting [4,8].

Typically, lame cows are visually detected by farmers based on abnormal locomotion or changes in behaviour and the identification of hoof lesions during routine trimming as the main clinical symptoms [3,9,10,11,12]. However, the subjectivity that these perceptions entail leads to an underestimation of the prevalence of lameness in a given herd [13,14]. To mitigate this issue, the locomotion scoring (LS) system first described by Sprecher, Hostetler, and Kaneene [15] has been gaining approval in large animal veterinary practice [9,10,16,17]. Although it is still a challenge at the farm level in many countries, promoting LS implementation is now considered useful worldwide from the perspective of sustainable livestock production and compliance with the Sustainable Development Goals (SDGs) and the One Health approach outlined by the United Nations (UN) [18,19].

This qualitative test is based on the degree of alteration in the biomechanics of the appendicular skeleton’s movement and its relationship with the spinal column’s line in standing and gait positions [15], consisting of a score from 1 (normal) to 5 (abnormal) that is given by an expert observer and can be supported by automated software [20]. Therefore, the LS system not only allows the early detection of foot disorders, but also aids in monitoring the prevalence of lameness, enables an accurate comparison of its incidence and severity, and can also be used to identify animals requiring functional trimming or treatment [9,11]. Moreover, it has proven useful in effectively predicting reproductive performance disorders since the time of Sprecher, Hostetler, and Kaneene’s findings [15] right up to the current day [21].

In general, it is known that the stress associated with lameness activates the hypothalamus–pituitary–adrenal axis and also affects the reproductive hypothalamus–pituitary–gonadal axis, as it is associated with low LH pulse frequency and delayed ovulation [22,23]. Moreover, a variety of negative effects of lameness on reproduction have been outlined in a wide range of studies, including an increase in days open, also denominated as calving to conception interval (CCI) [24]; the rise in the number of inseminations needed for conception [5,25,26,27,28]; and delayed ovarian cyclicity due to the presence of ovarian cysts [22,29,30]. Recently, lameness was associated with an increase in non-esterified fatty acids (NEFAs), which also contribute to delayed involution of the cervix and the formerly pregnant uterine horn, decreased ovulation rates, and increased atresia or cyst formation on day 50 postpartum [31].

The prevalence of lameness depends on a variety of intrinsic and extrinsic factors. Considering the present research, it is important to highlight that animals that produce high quantities of milk, such as the Holstein breed and its crosses, are more susceptible to lameness. The Jersey breed, originally from the British island of Jersey, is also recognised for its high milk yield and fat level, which are higher than those of other breeds in relation to their weight [32,33]. However, most of the studies on lameness and reproductive performance have been carried out on dairy breeds such as Holstein and Brown Swiss, with only some including a small proportion of Jerseys and crossbreeds in the sampled population [26,27,34,35,36,37,38]. Regarding extrinsic factors, humid and hot environments have been demonstrated to increase the prevalence of lameness in dairy cows [37,39,40,41] due to those conditions that can not only soften and damage hooves [42], but also trigger normal adaptive changes, such as an increase in total locomotor activity and a reduction in lying behaviour to minimise exposure to hot–humid surfaces [43]. Jerseys have been demonstrated to be much more successful in adapting to tropical conditions and their associated heat stress in comparison to other dairy breeds, such as Holstein [44,45,46]. Recently, a study about hoof lesions and fertility in lactating Jersey cows raised in humid continental locations was published [47]. However, it was not carried out under tropical conditions, and where other similar investigations are reported, none have addressed the Jersey breed specifically [25,48,49,50].

Therefore, the present study aims to investigate the impact of lameness on the reproductive postpartum efficiency of Jersey milking cows reared under hot–humid tropical conditions by evaluating the influence of LS on the reproductive postpartum variables such as the inseminations to conception, the CCI, and the conception rate.

## 2. Materials and Methods

### 2.1. Animals and Management

This study was conducted on a tropical dairy farm located in Bucay Canton in the Province of Guayas, Ecuador. The herd consisted of certified Jersey cattle, and the farm is identified as falling within the tropical ecological zone. Bucay Canton has a climate described as “humid equatorial”, is located at an altitude of 300–700 metres above sea level, and has an average annual temperature of 20 °C, average annual precipitation of 2000 mm, and relative humidity of 80% [51].

A total population sampling technique was used to recruit a homogenous target population of fifty-two (n = 52) Jersey milking cows aged 3 to 14 years, presenting a mean of 4.5 ± 0.4 lactations, average milk production of 2940 kg per lactation, and a weight of 300–450 kg with a body condition score of 2.5 to 3.7 using a 5-point (1–5) scale following the recommendations for Jersey’s cattle body condition evaluation [52,53]. There were no sampled animals left out of the study as a result of our exclusion criteria, which specified animals suffering common postpartum diseases in the immediately preceding calving, such as ketosis, milk fever, mastitis, left abomasum displacement, and dystocia [48].

All the selected cows were kept under similar management conditions throughout the study. They were housed in a rotational grazing-free stall, were milked two times daily using a mechanical milking system, had free access to pasture (mainly *Brachiaria decumbens*) and clean water, and were supplemented with pellets based on their individual requirements during milking.

A proven Timed Artificial Insemination (TAI) fertility protocol was performed after 60 days of a voluntary waiting period. To briefly summarise, this protocol specified a duration of seven days of treatment plus two more days for artificial insemination. The treatment included 2 mL of intramuscular oestradiol benzoate (Benzoato de Estradiol 100 mg, Fatro Von Franken, Buenos Aires, Argentina) with an intravaginal progesterone implant (Dispocel Max 1.2 g, Fatro Von Franken, Buenos Aires, Argentina) on day 1; 2 mL of prostaglandin (Dextrogenol 7.5 mg, Fatro Von Franken, Buenos Aires, Argentina); 1 mL of oestradiol cypionate (Sincro CP 100 mg, Ourofino, Brazil); and 1.5 mL of equine chorionic gonadotropin (Sincro eCG 6000 UI, Ourofino, Brazil), which were all applied intramuscularly on day seven following artificial insemination after 48–56 h with one 0.5 mL French straw of certified frozen–thawed semen containing 20 × 10^6^ sperm (ABS Global, Rio de Janeiro, Brazil). Pregnancy diagnoses were performed by ultrasound with a linear transducer of 5 MHz (Eco 2 Chison Medical Technologies Co., Ltd., Wuxi, China) 30 days after TAI and confirmed by rectal palpation after 70 days of TAI. For the animals of the study that did not conceive after the first TAI, the required TAI protocols were repeated until the establishment of a pregnancy. All data (day of calving, days of treatment, days and number of TAIs) were registered in the Genus Reproductive Management System (ABS Global, Delta, Minas Gerais, Brazil) to obtain the calving to conception interval (CCI) or days open, and the number of inseminations to conception.

### 2.2. Study Design

This research was performed with the consent of the Veterinary Medicine and Zootechnics Project Evaluation Committee of the University. It was carried out as a prospective observational cohort study with a non-experimental and cross-sectional design. All data were collected between 15 March 2019 and 15 March 2020.

To evaluate the patterns of movement in station and gait, all animals were visually examined by the second author, a trained locomotion scorer, on a horizontal, flat, dry, clean, and comfortable surface, following the recommendations of Huxley [38] and Van Hertem et al. [54]. Zinpro^®^ FirstStep^®^ Dairy Hoof Health and Management Program (Version 1.2.2; Zinpro Corporation, Eden Prairie, MN, USA) for LS evaluation was then used to verify the accuracy of the given score, resulting in the total coincidence of the scores obtained by visual LS scoring vs. the results of the automated LS scoring. Based on the LS system, a scale of 1 (non-lame), 2 (slightly lame), 3 (moderately lame), 4 (lame), or 5 (severely lame) was given to each animal [15]. Moreover, scores ≤ 2 were considered to correspond to subclinical lameness, and scores ≥ 3 to clinical lameness. Information about each animal regarding age, milk yield, lactation, body condition, and the reproductive postpartum parameters of inseminations per conception and the calving to conception interval, also known as days open, of each animal, was retrieved from the Genus Reproductive Management System (ABS Global, Delta, Minas Gerais, Brazil).

### 2.3. Statistical Analysis

Data were analysed using Rstudio (Version 1.3.1093; Integrated Development for R, RStudio, PBC, Boston, MA, USA) statistical software. LS was considered the independent variable, and reproductive variables associated with postpartum conception were considered the dependent ones. The conception rates of the entire herd and animals separated into groups of LS ≤ 2 and LS ≥ 3 were calculated using the ratio of pregnant cows to the total number of inseminations. Descriptive statistics of LS are expressed in frequencies, percentages, median, and mode, and quantitative data of the reproductive variables are expressed in mean ± SE. Before comparing the variables, the assumptions of normality and homoscedasticity were verified. Comparisons between LS and reproductive variables were made with a one-way Analysis of Variance (ANOVA) and a Tukey test in the case of compliance with the assumptions; otherwise, data were compared with the non-parametric Kruskal–Wallis test. Additionally, Spearman’s correlation test was performed to evaluate the relationship between LS and reproductive variables. A confidence level of 95% and a significance level of *p* < 0.05 were considered for all tests.

## 3. Results

### 3.1. Descriptive Statistics of LS and Reproductive Variables

**Figure 1:**
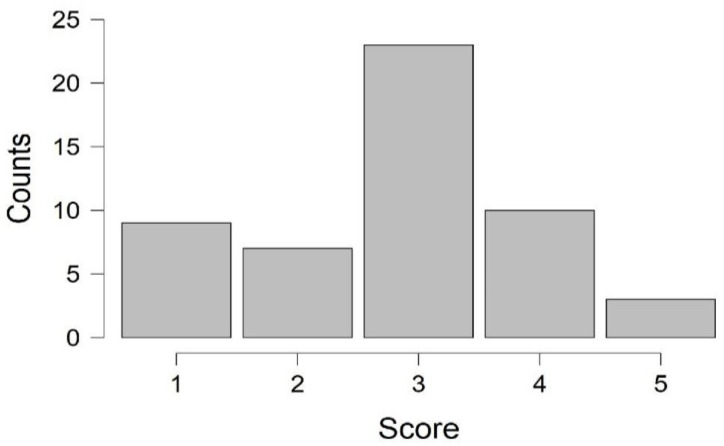
Distribution of LS in Jersey milking cows raised on a hot–humid tropical dairy farm.

A proportion of 82.6% (n = 43) of the total sampled cows (n = 52) presented lameness, with 13.4% (n = 7) being slightly lame (score 2) and 69.2% (n = 36) of cows exhibiting clinical lameness (scores ≥ 3) with a distribution of 44.2% (n = 23) for moderately lame (score 3), 19.2% (n = 10) for lame, and 5.7% (n = 4) for severely lame (score 5); meanwhile, 17.3% (n = 9) of cows were scored as sound (score 1). The herd showed negative asymmetry skewed to the left, with a median and mode score of 3, corresponding to moderate lameness (Figure 1).

Concerning reproductive variables, a conception rate of 21.8% for the entire herd (n = 52) was estimated. The group of non-lame and slightly lame cows (scores ≤ 2) had a conception rate of 45%, and clinically lame cows (scores ≥ 3) had a conception rate of 19.2%.

**Figure 2:**
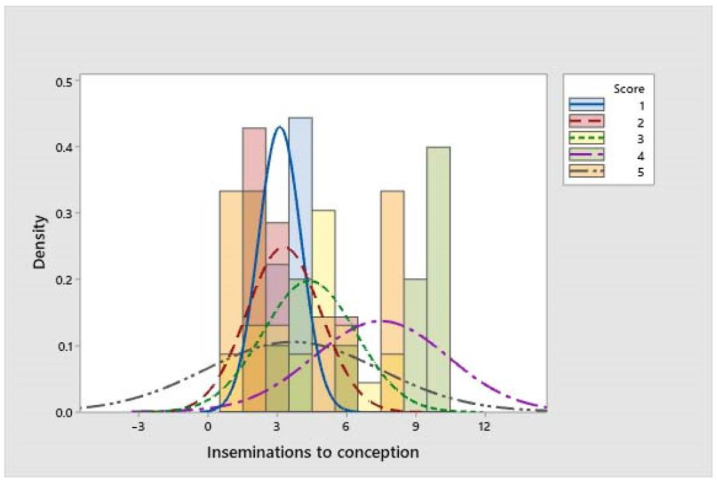
Behaviour of inseminations to conception data in Jersey milking cows with different LS (bars show the descriptive data of each score, and lines demonstrate the behaviour of the normal distribution, both based on the absolute frequencies).

A total number of 238 inseminations was registered for the herd. The scores that presented the lowest frequency were those corresponding to non-lame and slightly lame cows (scores 1 and 2). The scores with the greatest frequency were those for moderately lame and lame cows (scores 3 and 4). The latter showed the highest values of inseminations to conception (7.5 ± 0.9), but greater variation in the data was observed in this population. The score of 5 (severely lame cows) represented the most dispersed population, hence presenting the greatest variation in data in the study (Figure 2).

**Figure 3:**
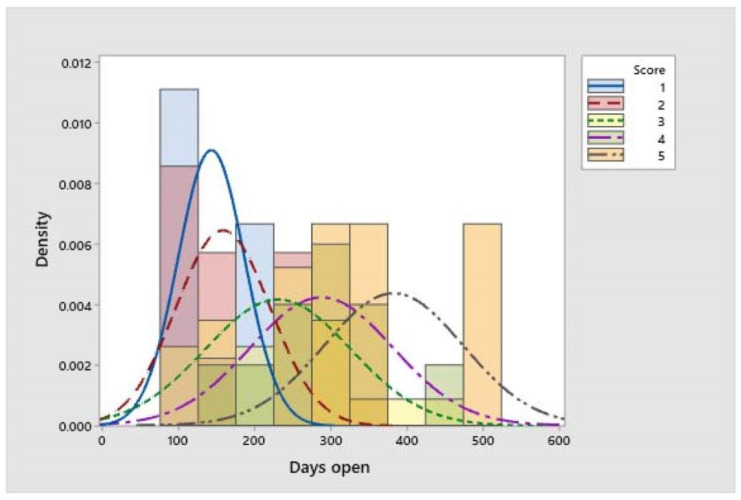
Behaviour of days open or calving to conception interval (CCI) in Jersey milking cows with different LS (bars show the descriptive data of each score, and lines demonstrate the behaviour of the normal distribution, both based on the absolute frequencies).

The herd’s CCI was 225.5 ± 14.3 days on average. The scores presenting greater frequencies were for moderately lame, lame, and severely lame animals (scores 3, 4, and 5), with the highest values for those found to be severely lame (383 ± 52.7). Non-lame and slightly lame cows presented the lowest frequencies with a similar distribution (142.9 ± 14.6 and 158.3 ± 23.3, respectively) (Figure 3).

### 3.2. Influence of LS on Reproductive Variables of Postpartum Conception

There were observed significant differences between LS and both inseminations to conception (*p* < 0.001) and CCI (*p* < 0.0001). Non-lame (score 1), slightly lame (score 2), and moderately lame (score 3) cows had a similar number of inseminations to conception, with a significant increase in lame animals (score 4). Severely lame (score 5) cows did not show significant differences from non-lame, slightly lame, moderately lame, and severely lame animals (scores of 1, 2, 3, and 4, respectively). Regarding CCI, there were no differences between non-lame, slightly lame, and moderately lame cows (scores 1, 2, and 3), but significant differences between lame and severely lame cows (scores 4 and 5) compared to scores 1, 2, and 3 were observed, although moderately lame and lame animals (scores 3 and 4) did not show differences in CCI (Table 1).

When splitting the LS independent variable, the group of cows with clinical lameness (scores ≥ 3) demonstrated a higher number of inseminations to conception and more days open than the groups of non-lame and slightly lame animals (scores ≤ 2), with *p* < 0.008 and *p* < 0.001, respectively (Table 2).

On the other hand, positive correlations between LS and both inseminations per conception and days open were observed. The correlation between LS and days open was moderate (r = 0.62; *p* ≤ 0.001). Additionally, a moderately positive correlation between both reproductive variables was observed (Table 3).

## 4. Discussion

Lameness in cattle is one of the most crucial issues affecting a veterinarian’s daily performance, since it compromises animal welfare and represents an important cause of economic losses in milk and meat production [38,55,56,57]. Evaluating lameness using the LS system has become an issue of great economic relevance in dairy herds [58,59,60]. In this study, a high proportion of Jersey cows presented lameness, with moderately lame animals representing almost half the dairy herd. Similar results have been reported with respect to the prevalence of subclinical and clinically lame cows [5,57,61]. However, a large variation among herds has been described, with ranges varying from one-third to more than half of the herd. These variations are mainly due to different interactions between the cows and their environments, such as the type of production system, breed, hardness of the hooves, ecology of the area, cow handling (for example, nutrition and the use of footbaths), frequency of hoof-trimming, flooring surface, and the skills of personnel responsible for identifying lame cows [48,49,50,62,63,64]. For instance, contrary to our results, lactation influenced the presentation of lameness in high-production Holstein cows housed in free-stall barns [37], and a very low incidence risk of 15% was reported for clinically lame cows in a seasonally breeding pasture-based system in New Zealand [34]. Moreover, the high proportion of cows suffering lameness in this study was certainly due to the constant levels of humidity in this tropical environment. Likewise, a prevalence of up to 76% of lameness was reported under similar conditions in Brazil [48]. Therefore, even though the Jersey breed has been demonstrated to adapt well to the tropics [44,45], and pasture-based systems seem to decrease the risk of lameness when compared with confinement systems [34,65], the hot–humid tropical conditions themselves might affect the prevalence of lameness, irrespective of how the animals are managed.

The consequences of lameness in fertility have been widely studied in many countries under different production systems and environmental conditions [2,5,22,28,29,34,38,61,64,66]. In the present study, all postpartum conception variables were related to lameness, and values increased within higher LS scores, confirming the negative effects of lameness as first outlined by Sprecher, Hostetler, and Kaneene [15]. Firstly, the conception rate of the herd was 21.8%, but when splitting scores between subclinical and clinical lameness, non-lame and slightly lame cows presented higher values than clinically lame cows (45% vs. 19.2%, respectively). On the other hand, the mean number of inseminations to conception was similar in non-lame, slightly lame, moderately lame, and severely lame cows, whereas animals scored as lame (score 4) required about 7.5 inseminations to conception. Additionally, a significant increase in inseminations to conception was seen when grouping the animals with subclinical and clinical lameness (3.1 vs. 5.1, respectively). Likewise, an increase in the number of required inseminations to conceive has been reported for lame Holstein cows [61,67]. For instance, one study described more than three inseminations to conception in cows with different causes of lameness, increasing to seven services to conception in cows presenting infectious pododermatitis and digital dermatitis, compared to non-lame animals that required 1.5 to 6 inseminations to conception [49,50].

The mean values in non-lame to moderately lame and severely lame cows were slightly higher than the normal number of 1.6 inseminations to conception established for dairy cattle [68], but the parameter was highly increased in cows scored as lame. It is to be noted that severely lame cows required a similar number of inseminations to non-lame, slightly lame, and moderately lame cows, but this could be explained by the low proportion of animals scored as severely lame (n = 4) and the high variance obtained; hence, the results could vary with a larger sample, although the proportions should remain similar.

Furthermore, non-lame and slightly lame cows had a CCI that ranged from 90 to 247 days in the present study, demonstrating a slight rise compared to the normal range defined in the literature [68]. However, moderately lame cows ranged from 77 to 429 CCI, and this range increased for lame and severely lame animals, from 131 to 472 days and 302 to 482 days, respectively. Taking the example established in the work of Sprecher, Hostetler, and Kaneene [15], which demonstrated the negative influence of lameness on days to first service and CCI, subsequent studies have strengthened the direct relationship of lameness with low fertility, principally by noting the increased number of inseminations, and therefore, CCI, with ranges that vary from 4 to 50 more days [5,21,26,28,29,30,34,50,61,69,70,71]. In addition, lameness has been reported to be the second-placed production disease for increasing days open after caesarean, and it is rated above other diseases such as endometritis, placental retention, dystocia, mastitis, and milk fever [72,73]. Although our results are similar to those described above, the higher increase in days open due to lameness stands out in this herd, probably due to the high proportion of clinically lame cows (LS ≥ 3).

Production diseases such as lameness influence the welfare and sustainability of dairy herds, resulting in long-term negative effects on milk yield, fertility, and the need to cull dairy cows [8,36,74]. This is a challenging aspect when considering dairy production in the tropics, where major factors such as economic restrictions, a lack of agricultural politics, and a lack of availability of robust scientific information already limit the ability to sustainably manage a dairy herd’s health. In these regions, cattle are also subjected to environmental conditions extremely different from those experienced in their native environments, such as hotter temperatures, greater solar radiation, and higher humidity [75,76,77,78]. Currently, the need to satisfy the increasing demand for animal-sourced foods and to reduce poverty in the tropics has led decision-makers to consider tropical livestock production as a relevant discipline within Animal Science. Keeping this in mind, region-specific strategies are being developed to contribute to sustainable livestock production within the conditions described above [18,76]. Among those strategies, early lameness detection by using the LS system and preventive management can contribute to improving animal welfare and sustainability at the farm level [79,80], since it represents a low-cost and easy-to-learn diagnostic tool that provides valuable information regarding all the stages of lameness [19,81]. This, in turn, could diminish the consequences related to postpartum conception in a hot–humid tropical environment and bring them more in line with those reported in non-tropical regions.

## 5. Conclusions

Fertility is an important factor to consider when discussing economic losses caused by foot disorders in livestock farming. Regarding the present study, certain limitations should be considered, such as the inclusion of a greater number of animals presenting different LS. This preliminary study indicates that even when subclinical lameness does not influence postpartum conception in Jersey cows raised under Ecuador’s hot–humid tropical conditions, special attention should be given to welfare and sustainability related to lameness in herds, since walking in less than 20% of cows was diagnosed as sound, and the parameters of inseminations to conception and days open were superior to those established in the literature for healthy animals. On the other hand, it is important to point out the high proportion biased overall postpartum conception variables for clinically lame cows. LS showed a positive correlation with both inseminations to conception and CCI; hence, higher scores were related to higher values of inseminations to conception and CCI. These results were also reflected in the decrease in the conception rate for the entire herd. Regular evaluation of the movement patterns of cattle through the LS system not only allows the early diagnosis of lameness and maintains healthy hooves, but also benefits the welfare and sustainable management of herds by promptly reducing discomfort due to lameness and maintaining the reproductive efficiency of postpartum conception in dairy cattle raised under hot–humid tropical conditions.

## Figures and Tables

**Table 1:** Average values of postpartum conception reproductive variables in Jersey cows presenting different LS expressed as mean ± SE.

Reproductive Variables	Non-Lame (1)	Slightly Lame (2)	Moderately Lame (3)	Lame (4)	Severely Lame (5)
Inseminations to conception	3.1 ± 0.3 ^a^	3.2 ± 0.6 ^a^	4.3 ± 0.4 ^a^	7.5 ± 0.9 ^b^	3.6 ± 2.1 ^ab^
Days open (CCI)	142.9 ± 14.6 ^a^	158.3 ± 23.3 ^a^	230.7 ± 19.9 ^ab^	287.9 ± 29.8 ^cb^	383 ± 52.7 ^c^

^a,b,c^ Means with different superscripts within the same row differ (*p* < 0.05).

**Table 2:** Average values of postpartum conception reproductive variables in Jersey cows presenting subclinical (scores ≤ 2) and clinical lameness (scores ≥ 3) in mean ± SE.

Reproductive Variables	Scores ≤ 2	Scores ≥ 3
Inseminations to conception	3.1 ± 0.3 ^a^	5.1 ± 0.4 ^b^
Days open (CCI)	149.6 ± 12.7 ^a^	259.2 ± 17.16 ^b^

^a,b^ Means with different superscripts within the same row differ (*p* < 0.05).

**Table 3:** Spearman’s correlations between LS and reproductive postpartum variables.

Reproductive Variables		Locomotion Score	Inseminations to Conception	Days Open
Inseminations to conception	Coefficient	0.390 **	—	
	*p*-value	0.002	—	
Days open (CCI)	Coefficient	0.620 ***	0.506 ***	—
	*p*-value	≤0.001	≤0.001	—

** *p* ≤ 0.01, *** *p* ≤ 0.001, one-tailed, for positive correlation.

## Data Availability

Data are contained within the article. The data that support this study are available in Mendeley Data, V1, doi: 10.17632/5xvj4t4b2t.1 (Accessed date: 23 March 2023).
